# Bioinformatics and Computationally Supported Redesign of Aspartase for β-Alanine Synthesis by Acrylic Acid Hydroamination

**DOI:** 10.1021/acscatal.4c05525

**Published:** 2024-12-30

**Authors:** Alejandro Gran-Scheuch, Hein J. Wijma, Nikolas Capra, Hugo L. van Beek, Milos Trajkovic, Kai Baldenius, Michael Breuer, Andy-Mark W. H. Thunnissen, Dick B. Janssen

**Affiliations:** † Chemical Biotechnology, Groningen Biomolecular Sciences and Biotechnology Institute (GBB), University of Groningen, 9747 AG Groningen, the Netherlands; ‡ Molecular Enzymology Group, Groningen Biomolecular Sciences and Biotechnology Institute (GBB), University of Groningen, 9747 AG Groningen, the Netherlands; § Baldenius Biotech Consulting, www.baldenius-biotech.com, 68159 Mannheim, Germany; ∥ BASF AG, GVF/E-A030, 67056 Ludwigshafen, Germany

**Keywords:** aspartase, β-alanine, bioinformatics, biocatalysis, computational design, hydroamination

## Abstract

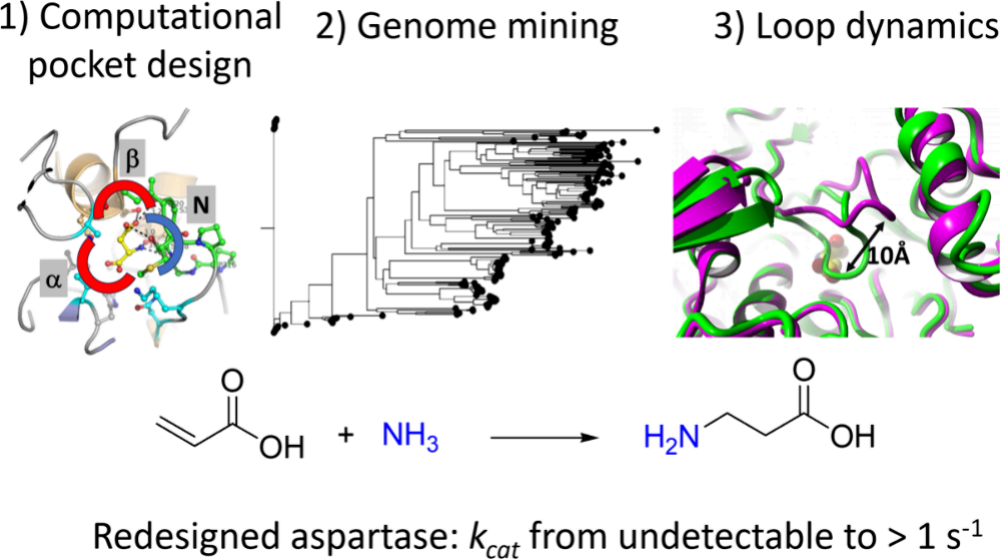

Aspartate ammonia lyases catalyze the reversible amination of fumarate to l-aspartate. Recent studies demonstrate that the thermostable enzyme from *Bacillus* sp. YM55–1 (AspB) can be engineered for the enantioselective production of substituted β-amino acids. This reaction would be attractive for the conversion of acrylic acid to β-alanine, which is an important building block for the preparation of bioactive compounds. Here we describe a bioinformatics and computational approach aimed at introducing the β-alanine synthesis activity. Three strategies were used: First, we redesigned the α-carboxylate binding pocket of AspB to introduce activity with the acrylic acid. Next, different template enzymes were identified by genome mining, equipped with a redesigned α-carboxylate pocket, and investigated for β-alanine synthesis, which yielded variants with better activity. Third, interactions of the SS-loop that covers the active site and harbors a catalytic serine were computationally redesigned using energy calculations to stabilize reactive conformations and thereby further increase the desired β-alanine synthesis activity. Different improved enzymes were obtained and the best variants showed *k*_cat_ values with acrylic acid of at least 0.6–1.5 s^–1^ with *K*_M_ values in the high mM range. Since the β-alanine production of wild-type enzyme was below the detection limit, this suggests that the *k*_cat_/*K*_m_ was improved by at least 1000-fold. Crystal structures of the 6-fold mutant of redesigned AspB and the similarly engineered aspartase from *Caenibacillus caldisaponilyticus* revealed that their ligand-free structures have the SS-loop in a closed (reactive) conformation, which for wild-type AspB is only observed in the substrate-bound enzyme. AlphaFold-generated models suggest that other aspartase variants redesigned for acrylic acid hydroamination also prefer a 3D structure with the loop in a closed conformation. The combination of binding pocket redesign, genome mining, and enhanced active-site loop closure thus created effective β-alanine synthesizing variants of aspartase.

## Introduction

Hydroamination of α,β-unsaturated carboxylic acids by Michael addition yields β-amino acids, which are components of various important bioactive compounds. An example is the conversion of acrylic acid to the nonproteinogenic amino acid β-alanine, an intermediate in the synthesis of pantothenic acid used for preparing coenzyme A (vitamin B5).^1^ Unfortunately, direct chemical addition of ammonia to acrylic acid is not practical since the required high process temperature leads to low yields and formation of side products.^2,3^ Consequently, the industrial route to β-alanine still involves the addition of ammonia to acrylonitrile followed by saponification to form calcium β-alaninate. Alternative routes to β-alanine, including enzymatic reactions, have been described.^4,5^ Unless integrated into biosynthetic pathways, such routes require multiple steps or lack atom efficiency, as is the case with β-alanine synthesis via α-decarboxylation of aspartic acid or oxidative deamination of 2,4-diaminobutyrate.^6,7^ Enzymatic conversion of acrylic acid to β-alanine would offer high atom efficiency and avoid side product formation, but no enzyme has been described to catalyze this reaction.

A noticeable enzyme that catalyzes double bond hydroamination is aspartate ammonia-lyase or aspartase (EC 4.3.1.1). In the 1970s, an immobilized whole-cell process for the continuous conversion of fumarate to aspartate was established in Japan by Tanabe Seiyaku Co.^8^ This showed that aspartase is an efficient biocatalyst suitable for commercial-scale applications. Whereas the reaction type is of interest for the synthesis of a diversity of amines and amino acids, aspartase is a very selective enzyme. Earlier attempts to modify its substrate range have met modest success. Asano et al.^9^ discovered a mutant of *E. coli* AspA (K327N) that accepts β-asparagine (l-aspartate β-amide) in the deamination reaction. Vogel et al.^10^ reported a directed evolution campaign targeting four residues at the substrate-binding pocket of *Bacillus* AspB. After screening 300,000 clones, a variant of *Bacillus* AspB was found that is active in crotonic acid amination, producing enantiopure (*R*)-2-aminobutanoic acid. In collaboration with B. Wu and co-workers, we have explored the use of computational enzyme redesign for obtaining AspB variants that catalyze hydroamination of different acrylic acid derivatives.^11^ The enzymes were used for kg-scale production of (*R*)-2-aminobutanoic acid, (*R*)-2-aminopentanoic acid, (*R*)-β-phenylalanine, and l-aspartate β-amide. Later work by Cui and co-workers^12^ showed that active aspartase derivatives with modified carboxylate- and amine-binding sites could be designed. This gave enzymes for the synthesis of amines from 9 different carboxylic acids and 14 different amines.

**Figure 1:**
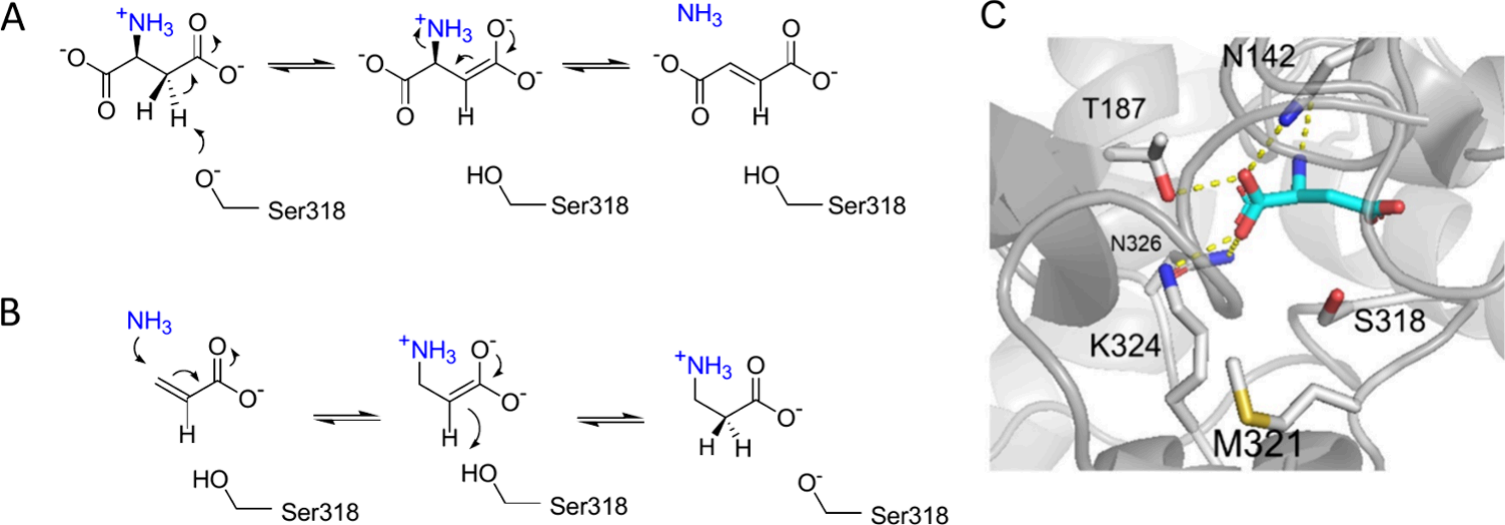
Mechanism of AspB and selection of target residues for mutagenesis. (A) Reaction mechanism of wild-type AspB, showing the role of the β-carboxylate group of l-aspartate in forming the enediolate reaction intermediate. (B) Hydroamination reaction of acrylate involves binding and conversion of a substrate missing the α-carboxylate group of l-aspartate. (C) Active site of AspB showing amino acids forming the α-carboxylate binding pocket targeted for mutagenesis (Thr187, Met321, Lys324, and Asn326).

There has been surprisingly little effort to develop enzymatic hydroamination of acrylic acid as a method to prepare commercially important β-alanine. The simplicity of the reaction and the avoidance of cosubstrates or cofactors make development of an aspartase that catalyzes this reaction an interesting protein engineering target, as described in patent applications.^13−15^ The latter describe mutations at sites that we engineered to introduce activity with substituted acrylic acids like crotonic acid.^11^ This earlier structure-based redesign work was based on the notion that mechanistically only the β-carboxylate functionality is expected to be essential for the amination/deamination reactions (Figure 1A,B). Accordingly, mutations that introduce activity with substrates missing the α-carboxylate group were sought by computation-based redesign at the 4 positions shaping the α-carboxylate binding pocket, including the replacement of a conserved lysine^11^ (Figure 1C). Furthermore, a conserved flexible loop, named SS-loop, that contains a two serine signature sequence plays an important role in substrate binding and catalysis. One of the serines acts as a general base in the deamination reaction by abstracting a proton from the C_β_ atom of the substrate, forming an enolate anion intermediate.^16^ For this, the SS-loop changes from an open conformation in the absence of substrate to a closed conformation that covers the active site in the presence of substrate. Studies on the functional importance of the SS-loop showed that in the open conformation the loop is disordered and suggested that the catalytic efficiency decreases as the disorder increases.^17^

Here, we report that aspartase can be redesigned to obtain a high activity with acrylic acid. First, we examined the redesign of the α-carboxylate binding pocket of AspB. After finding mutations that introduced β-alanine synthesis activity, we transferred these mutations to alternative template enzymes. The latter were selected by genome mining of databases with genome sequences of thermophilic bacteria. We also modified the interactions of the SS-loop in AspB and in the newly engineered variants. The best variants showed good β-alanine synthetic activity and were studied in different biotransformation reactions.

## Materials and Methods

### Active Site Redesign

Mutants were designed as described earlier,^11^ using PDB structure 3R6V. Briefly, Rosetta enzyme design^18^ was used to generate variants in which the hydrophilic α-carboxylate binding site was replaced by a site that can accept a hydrophobic substituent or just a hydrogen atom. The command line options were -enzdes -cst_predock -cst_design -detect_design_interface -cut1 0.0 -cut2 0.0 -cut3 8.0 -cut4 10.0 -cst_min -chi_min -bb_min -packing::use_input_sc -packing::soft_rep_design -extrachi_cutoff 1 -design_min_cycles 3 -ex1:level 4 -ex2:level 4 -ex1aro:level 4 -ex2aro:level 4. For this, 4 or more positions were varied, allowing for replacement by hydrophobic amino acids. In total, 26 designs were selected, of which 8 were reported earlier.

Rosetta^19^ and FoldX^20^ were also used to compute the difference between the effect of mutations close to the active site on the folding energy of the protein with the catalytic SS-loop in the closed conformation *vs* that energy with the loop in the open conformation. The options used for Rosetta were as follows: -ddg::weight_file soft_rep_design -ddg::iterations 50 -ddg::local_opt_only true -ddg::min_cst false -ddg::mean true -ddg::min false -ddg::sc_min_only false -ddg::ramp_repulsive false -ddg::opt_radius 8.0. These settings have earlier been referred to as the row 3 protocol.

### Mutant Construction and Enzyme Production

For mutant construction, we started with plasmids used for the production of AspB mutants B19, N5, F29, and P1 described earlier (Table 1).^11^ These templates are derived from pET21a, carrying an ampicillin resistance marker. The AspB coding sequences of 29 designs reported here were confirmed by DNA sequencing. The bioinformatic search was done using the sequence of AspB (NCBI accession BAA93044.1) as a template for sequence-driven genome mining using the NCBI server for BLAST searches. The DNA sequences of the selected putative aspartases were codon-optimized for *E. coli* and flanked by BsaI sites. The genes were cloned into pBAD-SUMO by Golden Gate assembly and transformed into chemocompetent *E. coli* NEB 10β cells.^21^

**Table 1:** Synthesis of β-Alanine by AspB Mutants

variant	T187	M321	K324	N326	number of times found^a^	β-alanine synthesis (mM)^b^	β-alanine synthesis (μmol/mg)^c^	specific activity (mU/mg)^d^	comments & source
AspB	T	M	K	N	n.a.^e^	0	0	–e	wild-type
A1	I	I	M	C	2185	57	12.7	49	top hit, this work
A2	I	M	F	C	1947	2	0.3	–	this work
A3	I	I	M	A	754	38	13.6	45	top hit, this work
A4	I	I	V	C	705	14	2.9	–	this work
A5	I	I	I	C	125	64	14.5	49	top hit, this work
A6	I	I	C	C	122	11	2.5	–	this work
A7	I	I	L	C	61	22	4.1	–	this work
A9	I	M	P	C	28	1	0.2	–	this work
A10	I	I	L	A	5	17	4.0	–	this work
A11	I	I	I	A	5	8	1.9	–	this work
A12	I	I	V	A	4	2	1.5	–	this work
A13	I	I	C	A	3	9	1.9	–	this work
A14	I	I	F	C	1	8	2.2	–	this work
A15	I	I	A	C	1	4	1.1	–	this work
A16	V	I	C	A	12	7	1.8	–	this work
A17	V	I	I	A	5	20	3.5	–	this work
B1	V	I	I	C	306	34	7.4	–	this work, Li et al.^11^
B10	V	I	M	C	705	31	8.9	–	this work, Li et al.
B2	V	I	C	C	648	4	1.2	–	this work, Li et al.
B5	V	I	M	A	327	8	1.4	–	this work, Li et al.
B7	V	I	V	C	1	4	1.7	–	Li et al.
B19	C	I	L	A	n.a	14	3.5	12	β-aminobutyric acid^11^
N5	T	I	N	C	n.a	1	0.2	–	β-asparagine^11^
P1	C	I	L	C	n.a	15	2.7	–	3-aminopentanoic acid^11^
Vogel	C	I	M	C	n.a	14	3.3	–	Vogel et al.^10^
Ai	V	I	L	C	n.a	43	9.6	35	intermediate, this work

a Number of times found in Rosetta redesign runs.

b Reactions were done with heat-treated aspartase variants. Values from 16 h reactions at 37 °C, 500 mM ammonia, and 250 mM acrylic acid. Data are from singular screening experiments.

c Synthesis of β-alanine expressed in μmol per mg of purified enzyme in the reaction mixture after 8 h.

d Activities were calculated over the initial 8 h. 1 U of activity corresponds to an amount of enzyme activity that catalyzes 1 μmol of product formation per min.

e n.a., not applicable; −, not determined.

Genes cloned in pBAD vectors were expressed using cells growing in Terrific Broth medium, supplemented with 50 mg/L ampicillin, and induced at OD_600nm_ 0.8 with 0.05% v/v l-arabinose followed by incubation for 48 h at 24 °C. Cells were harvested, and enzyme purification was performed as explained below. For better expression levels, selected variants were produced using the pET21a vector after transfer of the genes by Golden Gate assembly. Cells were grown in 5–10 mL of autoinduction medium containing 1% (w/v) tryptone, 0.5% (w/v) yeast extract, 0.33% (w/v) (NH_4_)_2_SO_4_, 0.71% (w/v) Na_2_HPO_4_, 0.68% (w/v) KH_2_PO_4_, 0.024% (w/v) MgSO_4_, 0.2% glycerol (v/v), 0.05% (w/v) glucose, 0.2% (w/v) lactose, and 50 mg/L ampicillin. For larger scale production, cells were grown in 50 mL of Terrific Broth medium with 50 mg/L ampicillin and induced at OD_600nm_ 0.6–0.8 with 1 mM IPTG for 24 h at 30 °C.

After cultivation, cells were harvested by centrifugation, and pellets were resuspended in 2 mL 50 mM Tris·HCl, pH 7.5, containing 2 mM MgCl_2_. Following sonication on ice, the lysate was heated in a water bath (60 °C, 1 h). The mixture was centrifuged (1 h, 18,500 × *g*, 4 °C) resulting in approximately 2 mL of cell-free extract devoid of heat-labile *E. coli* proteins. Protein concentrations were determined using the Bradford method, and purity was assessed by SDS-PAGE (Figure S1) and ImageLab (BioRad).

### Activity Assays

Reactions were set up using conditions as described for the hydroamination of *(E)*-2-pentenoic acid^11^ with a lowered concentration of acrylic acid since this compound might potentially inhibit the enzyme. Small-scale reactions were done at a 200 μL volume. Standard reaction mixtures contained 150 μL of 25 mM Na_2_HPO_4_, 20–250 mM acrylic acid, and 500 mM NH_3_, at pH 9.0. To this was added 50 μL of enzyme solution, giving a final protein concentration of 0.25–2.5 mg/mL. Reactions were run for 4–48 h at 25–70 °C in a flat-bottom 96-well plate with mixing.

Conversion was analyzed by HPLC (Figure S2). Samples were diluted 40-fold, and 100 μL of the resulting sample was mixed with 40 μL of 1 M NaHCO_3_ and 160 μL of DNFB solution (36.7 mM in acetone). After heating for 30 min at 60 °C, 80 μL of 1 M HCl was added and precipitates were removed by centrifugation.

HPLC conditions were as follows. Column: Nucleosil C18 5 μm (250 × 4.6 mm); temperature: 25 °C; eluent A, 0.1% formic acid in water; eluent B, acetonitrile; flow rate: 1 mL·min^–1^. DNFB-β-alanine detection with UV at 355 nm. Gradient: 15% B to 25% B in 6.5 min, 25% B isocratic until 24.5 min, 25% B to 50% B from 24.5 to 39 min, back to 15% B from 39 to 43.2 min. Retention time of DNFB-β-alanine: 23.3 min. A similar separation was achieved with shorter run times with isocratic elution: 45% A and 55% B. Retention time of DNFB-β-alanine: 5.8 min; detection range: 25–1300 μM. For comparison, β-alanine, d-alanine, and l-alanine were tested; all three compounds showed distinct retention times (Figure S2). The desired product was confirmed by ^1^H NMR (Figure S3).

Experiments for obtaining kinetic parameters were performed in duplicate, and statistical analysis was done using GraphPad Prism 9.3.0. For most enzymes, due to limited substrate solubility only lower limit values could be determined for *k*_cat_ and *K*_m_ and only catalytic efficiencies (*k*_cat_/*K*_m_) are reported. The obtained R^2^ correlation coefficients were >0.9 for all eight enzymes.

### Thermostability Assays

The thermostability of the aspartase variants was analyzed using the ThermoFluor method.^22^ Samples (20 μL) were prepared in a 96-well PCR plate. The samples contained 1 mg/mL of enzyme in 50 mM Tris·HCl buffer, pH 8.0, and 0.5% (v/v) commercial SYPRO orange fluorescent dye (5000-fold stock, Bio-Rad). The plates were heated from 20 to 99 °C, increasing the temperature by 1 °C per min using an RT-PCR instrument. After measuring fluorescence using excitation at 450–490 nm and a 560–580 nm emission filter, the apparent melting temperature was determined as the maximum of the derivative of the sigmoidal curve.

### Crystal Structure Determination

The protocol for protein isolation and crystal preparation is given in the Supporting Information. Crystals of the AspB variants, grown under different conditions and in the presence or absence of substrates, were sent for X-ray diffraction to the European Synchrotron Radiation Facility (ESRF) in Grenoble, France. Only crystals for unliganded AspB-6x and CcAsp-6x displayed diffraction. A suitable X-ray diffraction data set to 1.9 Å resolution was collected at beamline ID30A-1 for a crystal of AspB-6x grown in a crystallization solution containing 0.15 M NaCl and 28% PEG Smear Medium. At the same beamline, an additional data set to 3.1 Å resolution was collected for a CcAsp-6x crystal grown in crystallization solution containing 0.2 M MgCl_2_, 0.1 M Tris HCl pH 8.5, and 20% (w/v) PEG 8000. Prior to data collection, the crystals were briefly soaked in a cryoprotectant solution containing the mother liquor and 30% (v/v) glycerol and flash-cooled in liquid nitrogen.

Diffraction data were indexed and integrated using XDS,^23^ followed by scaling and merging with Aimless.^24^ The calculated Matthews coefficient of 2.48 Å^3^/Da and a solvent content of 50% indicated that the AspB-6x crystal contained two protein molecules in the asymmetric unit. The CcAsp-6x crystal also contained two protein molecules in the asymmetric unit with a Matthews coefficient of 3.21 Å^3^/Da and a solvent content of 62%. Initial phases were calculated using Phaser for molecular replacement, with a monomer of the AspB wild-type crystal structure as a search model (PDB code: 3R6V).^25^ Next, several iterations of manual model building with Coot^26^ and restrained refinement with REFMAC5^27^ were carried out to improve the structures. The quaternary structure (assembly) of the proteins was calculated and generated using PISA.^28^ A summary of the crystallographic statistics is given in Table S1. Coordinates and structure factors were deposited in the PDB with entry codes 8RJ0 and 8RJ1 for AspB-6x and CcAsp-6x, respectively.

Figures showing crystal structures and structure models were generated with PyMOL.^29^

### Calculation of AlphaFold Models

Tetrameric models of the AspB variants, including BbAsp-5x and StAsp-5x, were calculated with AlphaFold2 (version 2.3.1)^30^ using the AlphaFold-Multimer extension,^31^ as implemented on the Hábrok high performance computing cluster of the University of Groningen.

## Results and Discussion

### Introducing β-Alanine Synthesis Activity in AspB

We have previously described the redesign of the α-carboxylate binding pocket of AspB to obtain variants that catalyze hydroamination of substituted acrylic acids lacking the α-carboxylate functionality of aspartic acid.^11^ This gave enzyme variants that convert crotonic acid to β-aminobutanoic acid, (*E*)-2-pentenoic acid to β-aminopentanoic acid, fumaric acid monoamide to β-asparagine or (*E*)-cinnamic acid to β-phenylalanine. None of these enzymes were tested for the conversion of acrylic acid to β-alanine. To introduce this activity, we followed a computational approach similar to that described, using the Rosetta Enzyme Design application.

Rosetta calculations were done with the same variable positions as used previously, i.e., introducing more hydrophobic amino acids (Ala, Leu, Cys, Met, Phe, Trp, Gly, Val, Iso, or Pro) at positions Thr187, Met321, Lys324, and Asn326. There are two more residues (His188 and Asn142) that are also part of the carboxylate binding pocket, but these were not varied, because they also interact with the amine group of the product.

Promising variants were selected based on visual inspection and the following criteria: (1) the sum of the penalty energies for the above constraints should not exceed 20 Rosetta Energy Units (REU); (2) the active site must maintain the original hydrogen-bonding network around the β-carboxylate; (3) the introduced mutations do not result in large buried cavities; and (4) there should be no more than two unsatisfied hydrogen bond donors or acceptors in the designed active site.

The total number of unique variants found was 16; these are labeled A in Table 1. Interestingly, 14 of these featured Ile at position 187, instead of a Val in designs that were previously found for β-aminobutanoic acid.^11^ Comparing the structure to those of these earlier designs showed that the extra methyl group of the Ile fills a void that is created by shortening the substrate to β-alanine. In an attempt to generate a more diverse set of predicted variants, Rosetta calculations were repeated with no Ile allowed at position 187 and with the other positions restricted to amino acids found in catalytically active designs. This gave 6 more candidate designs, 5 of which had the same sequence of variants that were previously reported, which are labeled B in Table 1.^11^ In total, 22 designs were identified, of which 17 were new predictions, and 5 were the same as in the previous study on hydroamination of crotonic acid.

### β-Alanine Synthesis by Redesigned AspB Variants

The predicted variants were constructed by QuikChange site-directed mutagenesis and expressed in *E. coli* using either a pBAD-based or pET-based vector. Following purification by heat treatment of cell-free extracts, the synthesis of β-alanine from acrylic acid and ammonia was tested with product analysis by HPLC (Table 2). A few variants that were obtained earlier were included, including an enzyme designed for β-aminobutyric acid production (variant B19), the best variant for 3-aminopentanoic acid production (P1), and an enzyme for β-asparagine production (N5). The desired activity was found with several redesigned AspB variants. The earlier variants B19 and P1^11^ already showed some activity, but A1, A3, A5, B1, and B10 produced higher levels of β-alanine. Two mutants that were not computationally designed were also tested: the mutant designed by Vogel et al.^10^ showed only low activity but the variant obtained as an intermediate during stepwise mutagenesis, named AspB-Ai, showed activity.

**Table 2:** Selected AspB Homologues Identified by Genome Mining, the Cloning of their Mutated Genes, and Expression and Activity of the Mutated Proteins^a^

enzyme	organism	accession number of wild-type^b^	identity of wild-type to AspB^c^ (%)	*T*_m,app_ (°C)	β-alanine synthesis (mM)	β-alanine synthesis (μmol/mg)^e^
AspB	*Bacillus* sp. YM55–1	BAA93044.1	100	72	35	14
LaAsp	*Lysinibacillus acetophenoni*	WP_097148873.1	82.9	67	38	15
CcAsp	*Caenibacillus caldisaponilyticus*	WP_077616185.1	73.3	69	130	52
PcAsp	*Parageobacillus caldoxylosilyticus*	WP_061578972.1	72.2	50 and 72^d^	65	26
BbAsp	*Brevibacillus borstelensis*	WP_122958487.1	72.2	68.5	80	32
GAsp	*Geobacillus* sp.	WP_031409107.1	70.3	73	40	16
CdAsp	*Caldibacillus debilis*	WP_120667131.1	69.7	n.d.	20	8
StAsp	*Symbiobacterium thermophilum*	BAD39818.1	55.7	82	45	18

a All variants carry four mutations of the α-carboxylate binding site that correspond to the mutations that increased β-alanine synthesis in the A5 variant of AspB (T187I+M321I+K324I+N326C).

b NCBI accession number.

c Sequence identity with AspB as calculated using Geneious Prime.

d The thermogram profile showed 2 transitions.

e Synthesis was normalized by protein concentration and level of purity according to SDS-PAGE.

The mutants A1-A17 were named on the basis of their expected suitability according to the computational protocol (ranking by frequency of discovery by Rosetta). There seems to be a correlation between the observed activity and ranking. The mutants A1, A3, and A5 were ranked among the most promising for β-alanine synthesis and indeed appeared among the top performers when tested experimentally. Variants A2 and A9 all have methionine at position 321, which seems to completely abolish the activity with acrylic acid. In spite of the inclusion of a second computational library where Ile was forbidden at position 187, the three highest-scoring variants had an Ile at position 187.

For the best hits (A1, A3, A5, Ai, and B19), the reactions were repeated on a larger scale (1 mL). The β-alanine concentrations were again measured by HPLC, and the enzyme concentration was measured using Bradford. The reactions were followed over time, and the initial activities were calculated. The results suggested that variants A1 and A5 had the highest activity (Table 2).

### Genome Mining for Alternative Template Aspartases

This shows that the introduction of four mutations into AspB can introduce acrylate hydroamination activity. We expected that these mutations may have the same effect when introduced into other template enzymes, i.e. aspartases with high sequence similarity to AspB. Such alternative enzymes may well have active site geometries or features such as stability that influence their potential for acquiring β-alanine synthesis activity by mutations.

To identify alternative templates, we performed BLAST queries for AspB homologues in the NCBI database, searching genomes from thermostable organisms and metagenomes from hot springs. This gave 1450 potential AspB homologues which after removal of duplicates and enzymes not likely to be aspartases gave 97 candidates (Figure S4). After removing close homologues (>85% identity), this gave a final set of 24 sequences (Table S2). Expression of codon-optimized genes in which the mutations of variant AspB-A5 were included (corresponding to T187I+M321I+K324I+N326C) was tested with pBAD-based vectors and negatives were also tested with a pET-based expression vector.

Assays with heat-treated extracts identified 8 different expressed mutated aspartases that catalyzed β-alanine synthesis (Table 2). The best alternative enzymes for β-alanine synthesis were BbAsp, CcAsp, StAsp, LaAsp, PcAsp, CdAsp, and GAsp. These seven enzymes exhibited both good synthetic activity and high thermostability. BbAsp, which was expressed using the pET21a(+) vector, also showed a decent synthesis. One of these enzymes (CcAsp) displayed a synthetic yield higher than those of the A1 and A5 variants of AspB.

### Redesigning the Conformation of the Catalytic Loop

**Figure 2:**
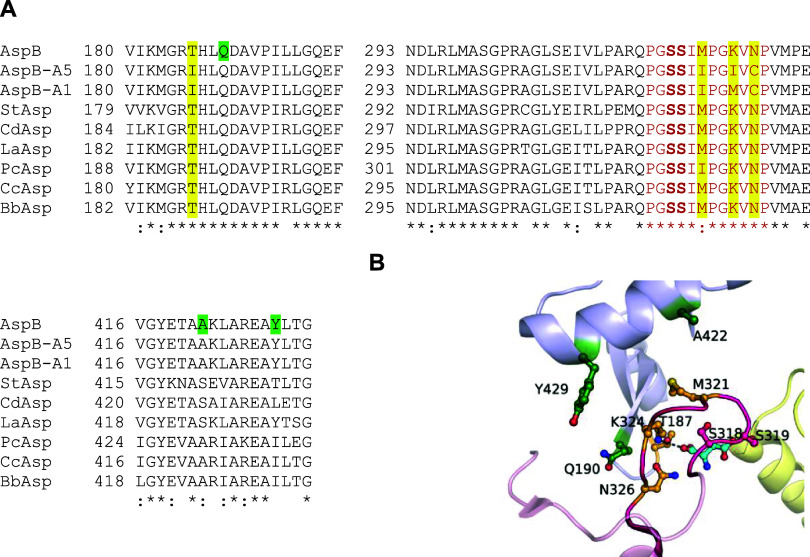
Active-site regions in selected aspartase variants. (A) Partial sequence alignment of AspB, AspB-A1, AspB-A5, and homologues that show β-alanine synthesis activity after introduction of the AspB-A5 carboxylate binding pocket mutations. The carboxylate binding pocket residues, their mutations in AspB-A1 and AspB-A5, and the corresponding positions in selected homologous aspartases are highlighted in yellow. Positions mutated to stabilize the loop in a closed conformation are marked green. The SS-loop residues are shown in red font; the loop is highly conserved among the AspB homologues. (B) Active site of substrate-bound AspB. The flexible loop is colored magenta. Residues forming the α-carboxylate binding pocket (orange) were replaced, including Lys324 that forms a salt bridge with the substrate (l-Asp, cyan). AspB has a composite active site: the three active site regions originate from different subunits in the tetramer, as indicated with different colors.

**Figure 3:**
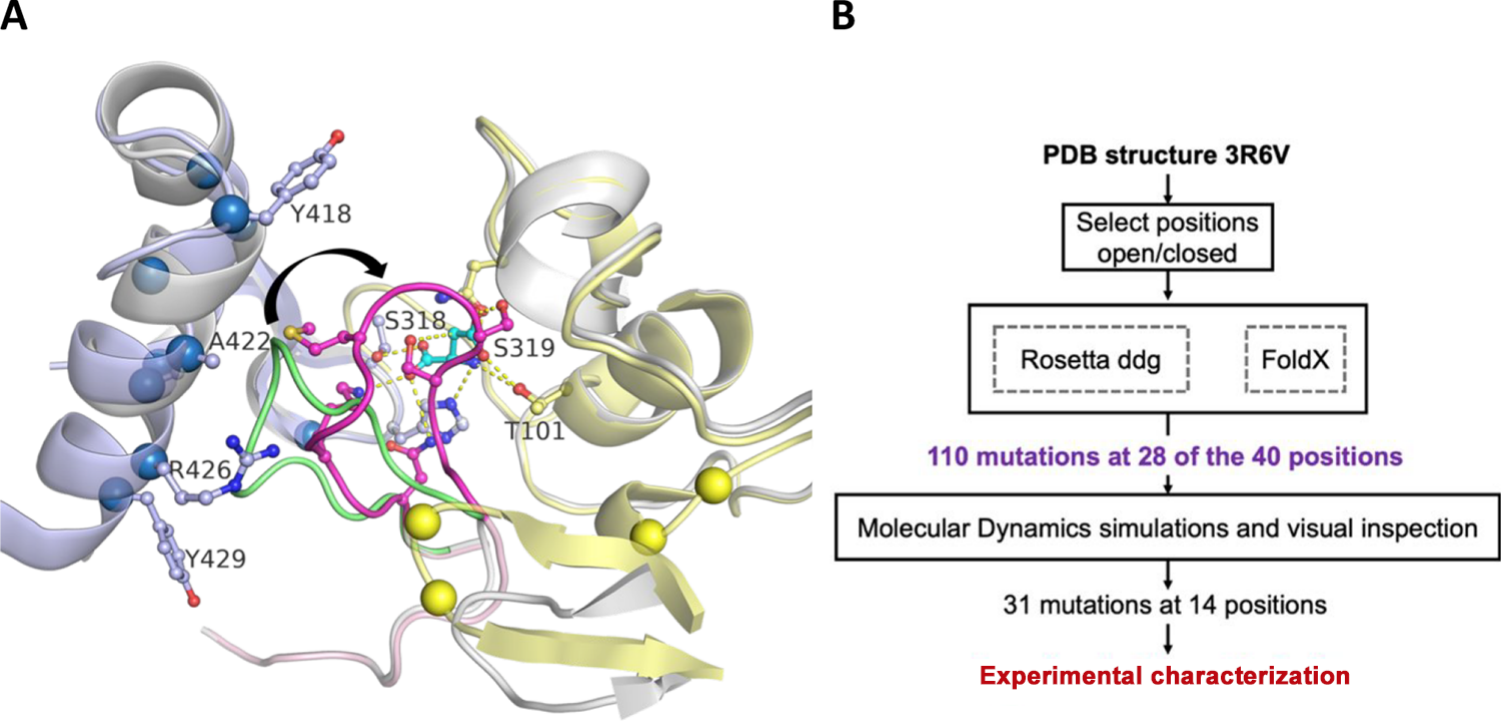
Design of mutations that promote the closed state of the SS loop. (A) Structure of a nonoccupied active site of chains B, C, and D (all in gray, except the SS-loop in green) aligned with the occupied active site (aspartate, in cyan) formed by chains A (blue, C-terminal domain), B (yellow), and chain C (pink, with the loop region harboring active site residues Ser318 and Ser319 in magenta). Loop closure (black arrow) brings active site residues including Ser318 and Ser319 in contact with the substrate at reactive positions. Dashes indicate H-bonds between enzyme and substrate. Large spheres indicate positions at which mutations were predicted to differentially stabilize the closed conformation or destabilize the open conformation. (B) Computational strategy for the discovery of mutations that promote the closed conformation. Rosetta and FoldX calculations were used to calculate the effect of point mutations on the closed and open conformations. Mutations predicted to mainly stabilize the closed form were subjected to MD simulations. MD-confirmed mutations were visually inspected and, if credible, tested experimentally.

The SS-loop in AspB is a crucial structural element in catalysis. It contains not only the conserved catalytic Ser318 but also residues that participate in forming the α-carboxylate binding pocket (Met321, Lys324, and Asn326) as well as a second conserved serine residue that is involved in catalysis (Figure 2). Structural studies indicated that this loop opens and closes during the catalytic cycle and that the closed conformation is required for catalysis.^17^ We considered that the energetics of loop closure might affect *K*_M_ and *k*_cat_ and that it may be disturbed by the mutations that removed the α-carboxylate pocket. Moreover, the remarkable specificity exhibited by AspB toward its native substrate may stem from the necessity of maintaining the active site in a specific closed conformation during catalysis. It has proven hard to obtain X-ray structures of the loop in its closed conformation, which indicates that it has a strong tendency to stay open and that closure is energetically enabled by substrate binding. For wild-type AspB, the poor conversion of amino acids other than aspartate may be related to ineffective loop closure or an improper conformation for catalysis. Conversely, since β-alanine is smaller than aspartate and lacks a carboxylate group, it can be expected that its potential binding energy is lower, and therefore the equilibrium in the mutant enzymes designed here may favor the open state too much, lowering the catalytic rate. We therefore decided to computationally screen for mutations that could stabilize the closed state relative to the open state (Figure 3).

Initially, all positions were considered that were at least 5 Å from the substrate and within 6 Å of the loop (residues 316–325) (Figure 3A). For these 40 positions, FoldX and Rosetta_ddg were used to computationally predict the effects of introducing all possible single-point mutations (except cysteine) on the stability of the open and closed states of the enzyme, using protocols described earlier.^32^ AI-based RoseTTAFold, which may serve the same purpose, became available after the design part of this project.^33^ If the stabilizing effect of a mutation was larger for the closed conformation (or the destabilizing effect was smaller) than that for the open conformation, that mutation was selected. For these calculations, the wild-type structure of AspB (PDB code: 3R6V) was used as it uniquely contains two subunits of AspB in the closed state and six subunits in the open state. Mutations were selected if they gave favorable predicted energy changes with either Rosetta or FoldX: maximally 7.5 kJ/mol destabilization of the free enzyme and a shift of at least 5 kJ/mol toward the closed state when comparing open and closed states. Mutations giving less favorable predictions were still included if they were suggested both by FoldX and Rosetta: maximally 15 kJ/mol destabilization and at least a 2.5 kJ/mol shift to the closed state with both programs. This resulted in the selection of 110 mutations at 28 positions (Figure 3B).

These 128 primary designs were subjected to MD simulations and visual inspection according to established FRESCO procedures and criteria.^32^ Mutants were eliminated if visual inspection revealed structural defects in the closed state, such as the presence of unsatisfied H-bond donors or acceptors. Additionally, mutants were eliminated if they caused changes in the conformation of the active site loop in the closed state. As a result, a total of 31 single mutations at 14 different positions were selected for experimental verification (Figure 3B).

The predicted closed-loop mutants were constructed in the AspB-A5 gene by QuikChange PCR and expressed in *E. coli* BL21(DE3) using pET21 in the usual way. Expression was very good in all cases, and the enzymes were isolated by heat-shock treatment. The partially purified proteins were examined by the standard method for β-alanine production from an acrylic acid. The AspB-A5 derivatives with modified loop contacts showed β-alanine formation at varying levels, mostly lower than those with AspB-A5. Gratifyingly, of the 30 mutants tested, 4 showed a slight improvement, while 2 showed significant enhancement of β-alanine synthesis (Figure S5). Levels of β-alanine produced varied from 24 to 40 μmol/mg enzyme in 24 h reactions (Table 3). From this, we conclude that the best mutations are A422S, A422E, Y429D, and Y429T. A comparison of experimental data with the calculations showed no clear trend for the distribution of activity of the mutants over the computed energy landscape (Figure S6), indicating that factors other than the energy-based equilibrium between open and closed states have a major effect on activity.

**Table 3:** Synthesis of β-Alanine by Closed-loop Mutants derived from AspB-A1 and AspB-A5^a^

	AspB-A5 pocket			AspB-A1 pocket^b^		
template and mutations	β-alanine (mM)	analytical yield (%)	TON^c^	β-alanine (mM)	analytical yield (%)	TON
AspB-derived template	8 ± 0.6	3.5	1800	53 ± 14	21	12,000
A422S	13 ± 0.2	5	3000	96 ± 8.2	38	21,300
A422E	15 ± 0	6	3500	99 ± 2.4	40	22,100
Q190I	3 ± 0.2	1	700	20 ± 4.2	8	4500
Y429D	26 ± 3	10	5800	89 ± 4.2	35	19,000
Y429T	14 ± 0.1	6	3100	54 ± 11	21	12,000
A422S+Y429D^b^				140 ± 2	56	31,000
A422S+Y429T				41 ± 4	16	9200
A422E+Y429D				120 ± 4	48	27,000
A422E+Y429T				89 ± 24	35	20,000

a Variants of AspB-A1 or AspB-A5 with the best mutations inducing the closed conformation of the SS-loop were produced in *E. coli* BL21(DE3) and purified by cell lysis, heating and centrifugation. The enzymes were compared by small-scale β-alanine synthesis reactions, which were carried out for 24 h at 37 °C in the presence of 250 mM acrylic acid and 500 mM ammonia and 0.5 mg/mL of isolated enzyme.

b The variant of AspB carrying the AspB-A1 pocket (T187I+M321I+K324M+N326C) and the loop mutations A422S+Y429D were used further and relabeled AspB-6x.

c TON, estimated total turnovers in mol of product per by mol of catalyst. Data represent averages of three reactions.

### Combining Mutations in Different Templates

The mutations predicted to stimulate closed-loop conformations and confirmed to enhance AspB-A5 acrylic acid hydroamination activity were also examined in the AspB-A1 background, which had higher synthetic activity than AspB-A5, as well as in the alternative templates described above with removed aspartate α-carboxylate binding pockets. Furthermore, some activity-enhancing mutations were tested in combination. Fortunately, this better performance of the AspB-A1 active site in comparison to AspB-A5 was retained when the most effective closed-loop mutations were introduced in Asp-A1 and three Asp-A1 mutants (A422S, A422E, and Y429D) showed improved β-alanine synthesis in comparison to the corresponding AspB-A5 variants.

To test combinations of closed-loop-inducing mutations, four double mutants of AspB-A1 were constructed (Table 3). Of these double mutants, three variants showed increased β-alanine production (1.7–2.7-fold higher) compared to the AspB-A1 variant and its derivatives with single mutations. These mutants retained the high thermostability of AspB (*T*_m,app_ = 69 °C). The best variant was AspB-A1+A422S+Y429D, and it was relabeled AspB-6x.

We also examined whether the BbAsp and CcAsp templates could be improved by introducing the A1 pocket instead of the A5 pocket and by mutations stimulating loop closure. StAsp was also included; two effective loop-closing substitutions detected with AspB-A1 (A422S and Y429T) are already present in its sequence. The resulting three variants equipped with the AspB-A1 pocket were well expressed and performed better in β-alanine synthesis than the corresponding variants with the A5 pockets (Table 4). These results identified the variants BbAsp-A1+A424E, CcAsp-A1+A424S+I431T, and StAsp-A1+S421E as the best enzymes for β-alanine synthesis. For the sake of brevity, they were renamed BbAsp-5x, CcAsp-6x, and StAsp-5x, respectively. Especially StAsp-5x looks attractive as it converted >90% of added acrylic acid to β-alanine. When the hydroamination reactions were tested with whole cells expressing the best aspartase derivatives as catalysts, again >90% conversion of acrylic acid to β-alanine could be obtained (Table S3).

**Table 4:** Comparison of Newly Discovered Enzymes and the Effect of Loop-stabilizing Mutations^a^

	template CcAsp			template BbAsp			template StAsp		
carboxylate pocket, loop mutation^b^^,^^c^	β-Ala (mM)	analytical yield (%)	TON	β-Ala (mM)	analytical yield (%)	TON	β-Ala (mM)	analytical yield (%)	TON
A5, none	55 ± 2	20	12,000	15 ± 3	6	3500	8.6 ± 2	3	1900
A1, none	84 ± 10	34	21,000	80 ± 21	32	20,000	83 ± 27	33	21,000
A1+ A″422″S	187 ± 7	75	40,000	71 ± 9	28	16,000	S present in wild-type		
A1+ A″422″E	52 ± 0.2	21	12,000	164 ± 2	65	36,000	234 ± 2	93	150,000
A1+A″422″E+ Y″429T″	not tested			127 ± 5	50	27,000	T present in wild-type		
A1+A″422″S+ Y″429″T	200 ± 15	80	44,000	not tested			S present in wild-type		
A1+ Y″429″D	33 ± 4	13	7500	17 ± 0.8	7	3800	60	24	13,000
A1+ Y″429″T	110 ± 11	44	24,000	29 ± 1.5	12	6500	T present in wild-type		
A1+ Q″190″I	66 ± 2	26	14,800	51 ± 11	20	11,000	37 ± 4	15	8300

a Reactions were carried out as described in Table 3.

b Reactions were carried out for 24 h at 37 °C in the presence of 250 mM acrylic acid, 500 mM ammonia, and 0.5 mg/mL of isolated protein as catalyst. TON, estimated total turnover per enzyme molecule. The number of replicates was 2.

c Mutations were introduced in the template at a position that corresponds to the AspB position given in quotation marks. The numbering is for the AspB sequence; for the corresponding residue numbering in other enzymes see Figure 2A.

### Characterization of the Best Enzymes

The four best enzymes obtained at this point were characterized further. Kinetic measurements were done by measuring initial rates of β-alanine formation at varying substrate concentrations of acrylic acid and 500 mM ammonia, pH 9.0 (Figure S7). For the A1 variants containing no further mutations, initial rates in millimoles of β-alanine formed per millimole of enzyme at 37 °C were up to 0.25 s^–1^ at 250 mM acrylic acid, the highest concentration tested. The insertion of the loop-closing mutations increased the initial rates for all 4 variants up to 0.6 s^–1^.

From these data, *k*_cat_ and *K*_M_ values were estimated, but for most enzymes, only lower limit values could be determined and only *k*_cat_/*K*_M_ were calculated (Table 5). The best enzyme by the *k*_cat_ criterion was BbAsp-A1+A424E, with a *k*_cat_ value exceeding 1.5 s^–1^. Most *K*_M_ values were >250 mM, indicating a low substrate affinity. For the AspB- and StAsp-derived variants, the *K*_M_ may be lowered by closed-loop-stabilizing mutations, but this cannot unequivocally be concluded from the data since only lower limits were found. A decrease in *K*_M_ is plausible, because of the design protocol. In the absence of a complete kinetic scheme, it cannot be excluded that other factors such as substrate entrance play a role and reduce the effect of loop closure on kinetics. The high *K*_M_ values will be of minor concern in industrial settings, where the use of high substrate loadings is preferable anyway.

**Table 5:** Kinetic Parameters of the Best Aspartase-derived Variants^a^

enzyme^b^^,^^c^	*k*_cat_ (s^–1^)	*K*_M_ (mM)	*k*_cat_/*K*_M_ (s^–1^·M^–1^)
AspB-A1	>0.22	>250	0.53 ± 0.03
AspB-A1+A422S+Y429D (AspB-6x)	>0.55	>250	1.9 ± 0.3
BbAsp-A1	>0.46	>250	1.6 ± 0.1
BbAsp-A1+A424E (BbAsp-5x)	>1.3	>250	4.2 ± 0.5
CcAsp-A1	0.49	180	2.7 ± 0.3
CcAsp-A1+A424S+I431T (CcAsp-6x)	0.74	230	3.2 ± 0.5
StAsp-A1	>0.24	>250	0.66 ± 0.12
StAsp-A1+A421E (StAsp-5x)	>1.2	>250	3.3 ± 0.3

a Due to the low affinity, no accurate *K*_M_ and *k*_cat_ values were obtained for most variants, only lower limits. Still, *k*_cat_/*K*_M_ values were calculated from the linear range of the direct Michaelis–Menten plots.

b Engineered enzyme variants had the carboxylate pocket replaced as in AspB-A1 (T187I+M321I+K324M+N326C) and additional closed-loop promoting mutations as indicated.

c Experiments performed in duplicate. The *R*^2^ values of the fits were >0.9 for all eight enzymes.

The kinetic parameters are comparable to those reported in the literature. Vogel and collaborators^10^ used directed evolution to obtain variants for the hydroamination of crotonic acid. The best mutant (T187C, M321I, K324M, and N326C) showed a *k*_cat_ of 0.09 s^–1^ and a *K*_M_ of 120 mM. In another study, Li and co-workers^11^ redesigned AspB for hydroamination of crotonic acid, (*E*)-2-pentenoic acid, fumaric acid, and (*E*)-cinnamic acid. The best variant showed a *k*_cat_ of 0.09 s^–1^ and a *K*_M_ of 900 mM for crotonic acid, while values for more bulky substrates ((*E*)-2-pentanoic acid, and (*E*)-cinnamic acid) were less impressive. A surface-engineered mutant showed a *k*_cat_ of 0.2 s^–1^ and a *K*_M_ of 430 mM for crotonic acid.^34^ These data illustrate that the four best variants described in this study exhibit kinetic parameters in the same range as or better than those of previously engineered variants for bulky substrates. The previous work has also demonstrated that hydroaminations catalyzed by aspartase variants can be performed on a preparative scale.^11^

### Crystal Structures of AspB Variants

**Figure 4:**
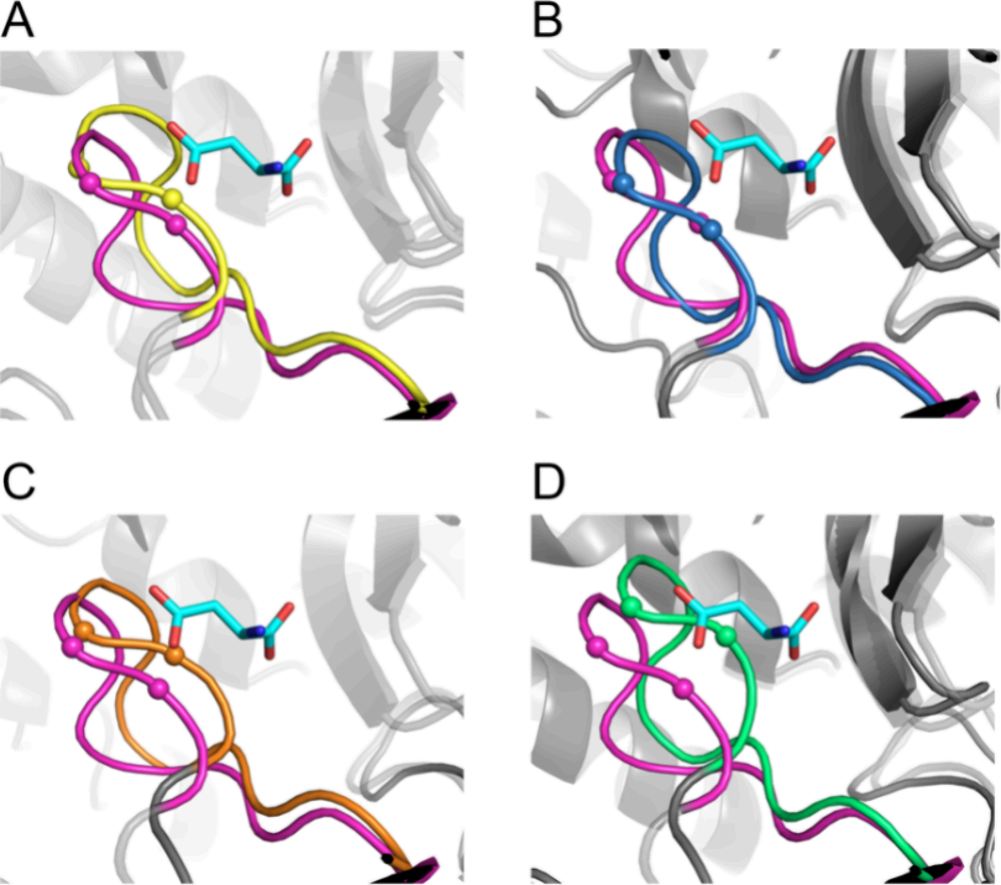
**C**omparison of the SS-loops in structures of the engineered variants and wild-type AspB. (A) Comparison of the crystal structures of AspB-6x (yellow) and wild-type AspB (magenta) with those of bound l-Asp (cyan). The SS-loops adopt very similar closed conformations, even though no substrate is bound in AspB-6x. (B–D) Similar comparisons were made with the crystal structure of wild-type AspB (magenta) of the crystal structure of CcAsp-6x (blue, B), an AlphaFold model of StAsp-5x (orange, C) and an AlphaFold model of BbAsp-5x (limegreen, D). The Cα-atoms of the two conserved serine residues in the SS-loops are shown as spheres.

To rationalize the effect of mutations that allowed for β-alanine synthesis, crystal structures of variants AspB-6x and CcAsp-6x were determined to 1.9 and 3.1 Å resolution, respectively (Table S1). Both variants were crystallized in an apo state: cocrystallization and crystal soaking experiments with acrylic acid, ammonia, or β-alanine did not yield a structure with a bound ligand. Both structures contained a protein dimer in the asymmetric unit, with functional tetramers formed via crystallographic symmetry. Analysis of the electron density maps confirmed the presence of the designed mutations at the active site and showed agreement between designed and observed SS-loop conformations, thus validating the designs (Figures 4 and S8).

A superimposition of the crystal structures of the two variants with the wild-type AspB structure (PDB code: 3R6V) revealed no substantial conformational differences except for the SS-loops. While the wild-type AspB crystal structure contains both open and closed SS-loops, unliganded AspB-6x and CcAsp-6x adopted the typical closed conformation observed previously only in the liganded active sites of wild-type AspB (Figure 4A,B). This similarity confirms the preference for a closed-loop conformation in variants with redesigned SS-loop interactions. The minor differences in side chain conformations of the two conserved SS-loop serines, compared to wild-type AspB with bound l-Asp, suggest that this closed conformation in the variants is suitable for accommodating acrylate and β-alanine in a position that is similar to that of bound l-Asp, with conserved interactions at the nonmutated β-carboxylic pocket.

**Figure 5:**
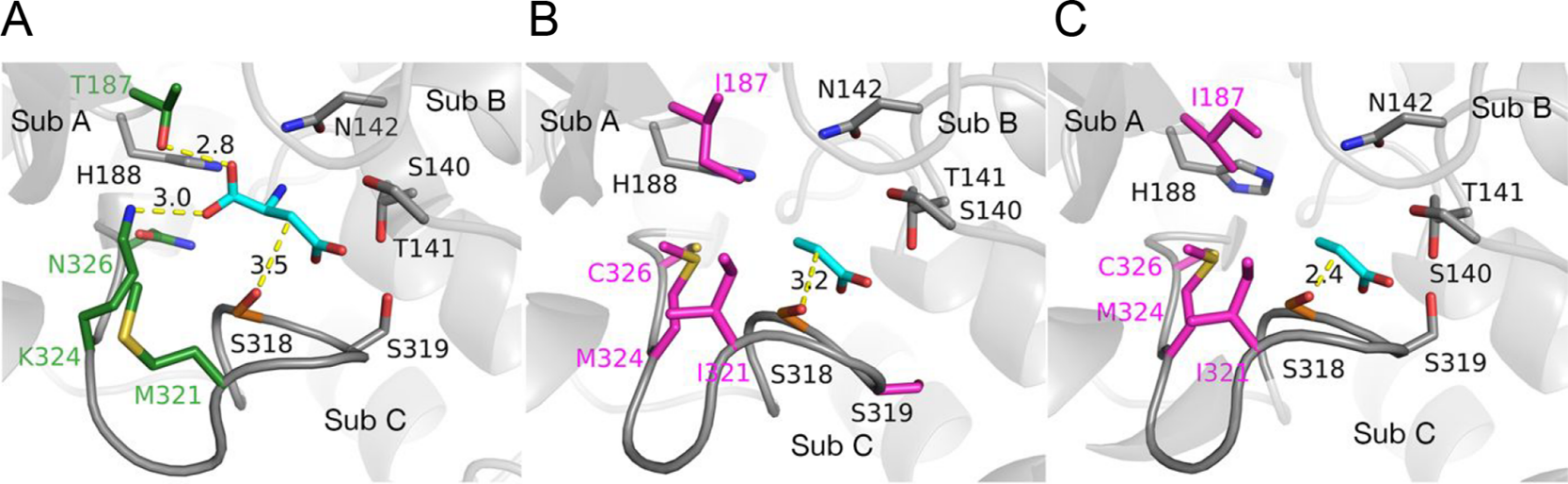
Comparison of the closed-loop active sites of wild-type AspB, AspB-6x, and CcAsp-6x. Distances are given in Å. (A) Active site of AspB, showing bound l-Asp with surrounding amino acid residues. Residues with green labels line the α-carboxylate binding pocket and were selected for mutagenesis. (B) Active site of variant AspB-6x showing the introduced mutations T187I, M321I, K324M, and N326C (magenta). Acrylate (cyan) is modeled in the active site by superimposition with l-Asp bound in the wild-type AspB structure. (C) Active site of the CcAsp-6x crystal structure bearing the same mutations as AspB-6x, shown in magenta. It appears that the catalytic serine loop is closer to the acrylate (cyan) modeled in the active site, in agreement with the higher activity compared to AspB-6x. The contacts between the hydroxyl group of catalytic Ser318 (orange) and the reactive carbon atom of the aspartate and acrylate compounds are shown as yellow dashed lines.

To examine this further, acrylate was modeled in the active sites of AspB-6x and Cc-Asp-6x by retracing from the β-carboxylate of l-Asp, as bound in wild-type AspB (Figure 5). The modeled complexes revealed that the carboxylate group of acrylate and the β-carboxylate group of aspartate may interact similarly in the conserved β-carboxylate binding pocket. Moreover, the terminal C3 methylene group of acrylate may occupy a hydrophobic pocket formed by the side chains introduced by T187I, M321I, K324M, and N326C.^11^ The contacts between the C3 carbon of the modeled acrylic acid and the side chains of Ile187 and Met324 occur at interatomic distances of about 4 Å, which would be optimal for hydrophobic binding. The side chains of Cys326 and Ile321 are positioned a bit farther away at distances of 4.5–5 Å. Importantly, the C2 carbon of acrylate is located in close proximity to Ser318-OH (Ser320 in CcAsp-6x), at a distance of ∼3 Å, which is suitable for its deprotonation during the reaction that results in the formation of β-alanine. In the CcAsp-6x variant, the SS-loop adopts a slightly different conformation as in AspB-6x, allowing the active site residues, including the catalytic serine, to more closely approach the substrate. This subtle difference in the SS-loop conformation may perhaps be related to the higher activity of the CcAsp-6x variant.

To explain the preference for a closed SS-loop conformation, we examined the AspB-6x and CcAsp-6x structures around the loop region in more detail (Figure 5). This revealed that the four active site mutations that introduce acrylic acid hydroamination activity form various favorable interactions with neighboring active site residues. In AspB-6x, the introduced Ile321 interacts with Val193, while Met324 makes hydrophobic contacts with Ala192, Gln190, and the mutated Ile187. Additional hydrophobic contacts are formed between Cys326 and Val328. Similar interactions occur in CcAsp-6x. Thus, the mutations that remove the charged binding pocket for the α-carboxylate group of aspartate may also have a stabilizing effect on the closed SS-loop conformation. In contrast, the activity-enhancing A422S and Y429D mutations that were designed to promote the closed conformation of the SS-loop in AspB-6x are positioned in helix α16 of the small C-terminal domain of an adjacent subunit. This domain is located close to the SS-loop and influences its orientation.^17^ In the crystal structure of wild-type AspB, the open conformation of the SS-loop at unliganded active sites is stabilized via hydrophobic interactions with C-terminal domain residues Ile406, Tyr418, Ala421, Ala422, Ala425, and Tyr429.^17^ Mutations A422S and Y429D disrupt this hydrophobic cluster and thus will destabilize the open-loop conformation and shift the equilibrium toward the closed-loop conformation (Figure S9). A similar destabilizing effect on the open SS-loop conformation can be rationalized for the A424S and I431T mutations in CcAsp-6x.

### AlphaFold Models of BbAsp-5x and StAsp-5x

Since no X-ray diffracting crystals were obtained for BbAsp-5x and StAsp-6x, we used AlphaFold-Multimer^31^ to predict their tetrameric structures (Figure 4C,D). Interestingly, the AlphaFold structures of BbAsp-5x and StAsp-6x are highly similar to the crystal structures of AspB-6x and CcAsp-6x, also showing a closed SS-loop conformation. The C-terminal domain mutations in BbAsp-5x and StAsp-5x (A424E and S421E, respectively) introduce a negative charge at the protein surface at a location that is equivalent to that of Ala422 in wild-type AspB. Thus, the AlphaFold models of BbAsp-5x and StAsp-6x point to similar mechanisms for improvement of the amination activity of these variants. However, we are aware that care should be taken when using AlphaFold models because of the uncertainties in predicted side chain orientations and loop orientations.^35^ These uncertainties may compromise their use as a template for docking substrates.^36^ Therefore, the conjecture that the enhanced catalytic efficiency of variants BbAsp-5x and StAsp-5x stems from the same effects as those proposed for AspB-6x and CcAsp-6x remains speculative.

## Conclusions

We have obtained aspartase-derived robust biocatalysts that are conveniently produced in *E. coli* and can be isolated by cheap freeze–thaw lysis and heat treatment with the removal of debris by centrifugation. The resulting enzyme preparations catalyze reasonably rapid synthesis of β-alanine from acrylic acid, and by choice of the right pH close-to-full conversion can be achieved if ammonia is added in excess.

The desired aspartase-derivatives were obtained by integrating three approaches: (i) removal and redesign of the α-carboxylate binding pocket for the natural substrate l-aspartic acid, (ii) bioinformatics-based selection of aspartase-homologues to serve as new templates, and (iii) computationally-supported protein engineering to stimulate the catalytically relevant loop to adopt the closed conformation required for catalysis.

Crystallographic analysis showed that the predicted Rosetta structures are in good agreement with the adopted 3D crystal structures, with no major deviations in backbone or side-chain geometry. The inserted mutations allow for formation of hydrophobic contacts with acrylic acid and for positioning of this new substrate in the active site, both in the AspB variant AspB-6x and in the homologue CcAsp-6x. The latter was generated by the transplantation of activity-enhancing mutations into a homologous aspartase discovered after genome mining.

The catalytically required closed conformation of the SS-loop, which is also found in the aspartate-bound structure of the wild-type enzyme, was observed in the crystal structures of the redesigned variants, even though no substrate was bound. The differences in the catalytic efficiency are likely connected to a higher preference for loop closure. This higher preference for loop closure may include two factors: (i) more stabilization of the closed SS-loop conformation due to tighter interactions and/or (ii) more destabilization of the open SS-loop conformation, triggered by the local redistribution of surface charges in the C-terminal domain of a subunit that flanks the loop. The activity-enhancing effect of the C-terminal domain mutations supports the notion that the enzyme engineering of a flexible active site should focus not only on stabilizing reactive conformations but also on destabilizing unreactive conformations.

## Supporting Information Available

Information on enzyme isolation and preparation of crystals and details on HPLC protocols, genome mining, and enzyme characterization (PDF)
