# Supplementary material for: Bioinformatics and Computationally Supported Redesign of Aspartase for β-Alanine Synthesis by Acrylic Acid Hydroamination
Source: ACS Catal. 2024 Dec 30;15(2):928–38. doi: 10.1021/acscatal.4c05525 (PMC11744663; doi:10.1021/acscatal.4c05525)
Supplement: Supplementary file 1 — cs4c05525_si_001.pdf [file cs4c05525_si_001.pdf]

Supporting Information

Bioinformatics and computationally supported redesign of aspartase for β-alanine synthesis by acrylic acid hydroamination

Alejandro Gran-Scheuch,[1] Hein J. Wijma,[1] Nikolas Capra,[1] Hugo L. van Beek,[1] Milos Trajkovic,[2] Kai Baldenius,[3] Michael Breuer,[4] Andy-Mark W.H. Thunnissen[1], Dick B. Janssen[1,*]

[1] Chemical Biotechnology, Groningen Biomolecular Sciences and Biotechnology Institute (GBB), University of Groningen, 9747 AG Groningen, The Netherlands
[2] Molecular Enzymology Group, Groningen Biomolecular Sciences and Biotechnology Institute (GBB), University of Groningen, 9747 AG Groningen, The Netherlands
[3] Baldenius Biotech Consulting, www.baldenius-biotech.com, 68159 Mannheim, Germany
[4] BASF AG, GVF/E-A030, 67056 Ludwigshafen, Germany

*Corresponding author. E-mail: d.b.janssen@rug.nl

Crystallization

For protein crystallography, the enzyme variants AspB-6x, BbAsp-5x, CcAsp-6x and StAsp-5x as obtained with the protocol mentioned above were further purified by size exclusion chromatography using a Superdex S200 13/30 column (Cytiva, Sweden) on an ÄKTA FPLC system. Samples were loaded onto the column previously equilibrated with 25 mM Tris·HCl, pH 9.0, and 100 mM NaCl. The proteins eluted in a single peak corresponding to a molecular weight of ~180 kDa, consistent with the presence of tetramers. Sample purity was assessed by SDS-PAGE, showing a purity of at least 95%. Dynamic Light Scattering (DLS) analysis revealed that the samples were sufficiently monodisperse and confirmed the presence of tetramers.

Prior to crystallization screenings, protein samples were concentrated to 10 mg/ml in 25 mM Tris·HCl, pH 9.0, and 100 mM NaCl and aliquots of the protein were incubated for 30 min with 0.1 M acrylic acid and 0.2 M NH_3, pH 9.0. Crystallization screens were carried out at 20º C using the sitting drop method with different sparse-matrix crystal screens and a Mosquito crystallization robot. Drops were dispensed in MRC-SD2 plates by mixing protein (apo or substrate-incubated) and reservoir solutions at two different ratios (0.75:1.25 and 1.25:0.75) to a final volume of 200 nL. For all variants, small single crystals appeared after 4 days at a few different crystallization conditions. Only the crystals of AspB-6x and CcAsp-6x produced suitable X-ray diffraction. The best crystals for AspB-6x grew in BCS screen C3 (Molecular Dimensions, UK), containing 0.15 M NaCl and 28% PEG Smear Medium, while for CcAsp-6x crystals grew in the JCSG screen D6 (Molecular Dimensions), containing 0.2 M $MgCl_2$, 0.1 M Tris, pH 8.5, and 20% (w/v) PEG 8000. Optimization attempts were undertaken to obtain larger crystals by varying protein concentration, temperature, salt and precipitant concentration, crystallization buffer concentration and pH. Diffraction from crystals thus obtained did not differ significantly from that with crystals obtained from the screenings. Some of the crystals obtained in the absence of acrylic acid and NH_3 were used for soaking experiments with β-alanine at concentrations varying between 20 mM and 100 mM.

Table S1. Crystallographic data collection and refinement statistics

**Data collection**	**AspB-6x**	**CcAsp-6x**
Beamline	ESRF ID30A-1	ESRF ID30A-1
Wavelength (Å)	0.9654	0.9654
Space group	$P2_122_1$	$P3_221$
Unit cell dimensions		
a,b,c (Å)	75.9, 99.0, 136.4	164.2, 164.2, 86.8
Resolution range (Å)[a]	49.5 -1.9 (1.94-1.90)	47.4 -3.1 (3.31-3.10)
Total observations[a]	370687 (21304)	363317 (63226)
Unique reflections[a]	80977 (4403)	24734 (4419)
$\langle I/\sigma \rangle$[a]	10.7 (1.1)	8.9 (1.4)
$CC_{(1/2)}$[a]	0.998 (0.388)	0.996 (0.578)
Completeness (%)[a]	99.3 (99.5)	99.9 (100.0)
**Multiplicity**	4.6 (4.8)	14.7 (14.3)
R_{meas} (%)[a]	0.084 (1.586)	0.377 (2.428)
**Refinement**		
R-factor/R_{free} (%)[b]	0.17/0.21	0.20/0.26
Number of non-H atoms		
protein	7149	7096
water	214	
Average B (Å^2)	45.7	85.9
RMSD		
Bond lengths (Å)	0.014	0.008
Bond angles (°)	2.1	2.7
Ramachandran plot		
% favored, outliers	98.5, 0.7	95.1, 0.3
**Rotamers, %outliers**	2.7	3.3
**Molprobity score**	1.39	2.15
**PDB entry**	8RJ0	8RJ1

[a] Values in parentheses correspond to the highest resolution shell.

[b] R_{free} is calculated as R-factor using 5% of all reflections randomly chosen, which were excluded from structure refinement.

Table S2. Selected AspB homologs identified by genome mining. The accession number corresponds to the NCBI accession code. Sequence identity with AspB was calculated using Geneious Prime. The optimal growth temperatures are adopted from reported work.

Enzyme	Accession number of wild-type	% identity to AspB	Source of organism	Optimal growth temperature [°C]
LaAsp	WP_097148873.1	82.9	*Lysinibacillus acetophenoni*	40 °C[a]
CcAsp	WP_077616185.1	73.3	*Caenibacillus caldisaponilyticus*	40-65 °C
PcAsp	WP_061578972.1	72.2	*Parageobacillus caldoxylosilyticus*	65 °C
BbAsp	WP_122958487.1	72.2	*Brevibacillus borstelensis*	45-70 °C
GtAsp	WP_100660238.1	70.9	*Geobacillus thermodenitrificans*	60-70°C
GAsp	WP_031409107.1	70.3	*Geobacillus sp.*	55-65 °C
CdAsp	WP_120667131.1	69.7	*Caldibacillus debilis*	50-70 °C
TbAsp	HHX23930.1	62.1	*Thermoanaerobacterales bacterium*	n.r. [b]
StAsp	STH833	55.7	*Symbiobacterium thermophilum*	60 °C
TtcAsp	WP_028992227.1	53.2	*Thermoanaerobacter thermocopriae*	60 °C
TAsp	WP_096231405.1	53.0	*Thermoanaerobacterium sp. RBIITD*	50-55 °C
PtAsp	BAF59162.1	52.8	*Pelotomaculum thermopropionicum SI*	55 °C
TtAsp	WP_150201177.1	52.4	*Thermoanaerobacterium thermosaccharolyticum*	60-65 °C
CfAsp	WP_073341503.1	52.4	*Caldanaerobius fijiensis*	60-63 °C
TtoAsp	WP_084665725.1	51.6	*Thermanaeromonas toyohensis*	70 °C
CpAsp	WP_026486371.1	51.3	*Caldanaerobius polysaccharolyticus*	65 °C
DkAsp	Desku_2319	50	*Desulfofundulus kuznetsovii DSM 6115*	60 °C
Masp	WP_054938070.1	49.9	*Moorella sp.*	40-60 °C
AdAsp	WP_015739228.1	49.5	*Ammonifex degensii*	70 °C
CsAsp	ERM91146.1	47.6	*Caldanaerobacter subterraneus subsp. yonseiensis KB-1*	75 °C
TmAsp	THEMA_02505	40.3	*Thermotoga maritima*	80 °C
MhAsp	WP_106004783.1	49.5	*Moorella humiferrea*	65 °C
DtAsp	WP_027717497.1	49.2	*Desulfovirgula thermocuniculi*	70 °C
TaAsp	WP_123931054.1	48.7	*Thermodesulfitimonas autotrophica*	65 °C

[a] Described to show solvent tolerance.

[b] n.r., Not reported. Organism isolated from an anaerobic digestor.

Table S3. Conversions using whole cells as catalysts[a].

	Analytical yield (%)								
**Temperature**	**37 °C**			**55 °C**			**70 °C**		
**Time**	**4 h**	**24 h**	**48 h**	**4 h**	**24 h**	**48 h**	**4 h**	**24 h**	**48 h**
AspB-6x	28 ± 6	99 ± 3	99 ± 5	89 ± 12	99 ± 4	99 ± 5	60 ± 1	97 ± 3	94 ± 2
BbAsp-5x	55 ± 4	99 ± 2	95 ± 5	97 ± 4	99 ± 2	99 ± 2	n.d.	n.d.	n.d.
CcAsp-6x	22 ± 4	80 ± 15	94 ± 5	74 ± 2	99 ± 3	99 ± 2	n.d.	n.d.	n.d.
StAsp-5x	15 ± 2	90 ± 3	84 ± 9	48 ± 1	98 ± 9	91 ± 10	43 ± 7	80 ± 6	85 ± 3

[a] The best variants obtained in this work were produced in *E. coli* BL21(DE3). Cells were resuspended in 1/10 of the culture volume in 50 mM Tris-HCl, pH 9.0. Synthesis of β-alanine synthesis was tested in duplicate in reaction mixtures incubated for 4, 24 or 48 h at 37°C, 55°C or 70°C and containing 250 mM acrylic acid and 500 mM ammonia in 25 mM Na_2HPO_4, pH 9.0, and 25% (v/v) resuspended cells. Data are averages of duplicates.

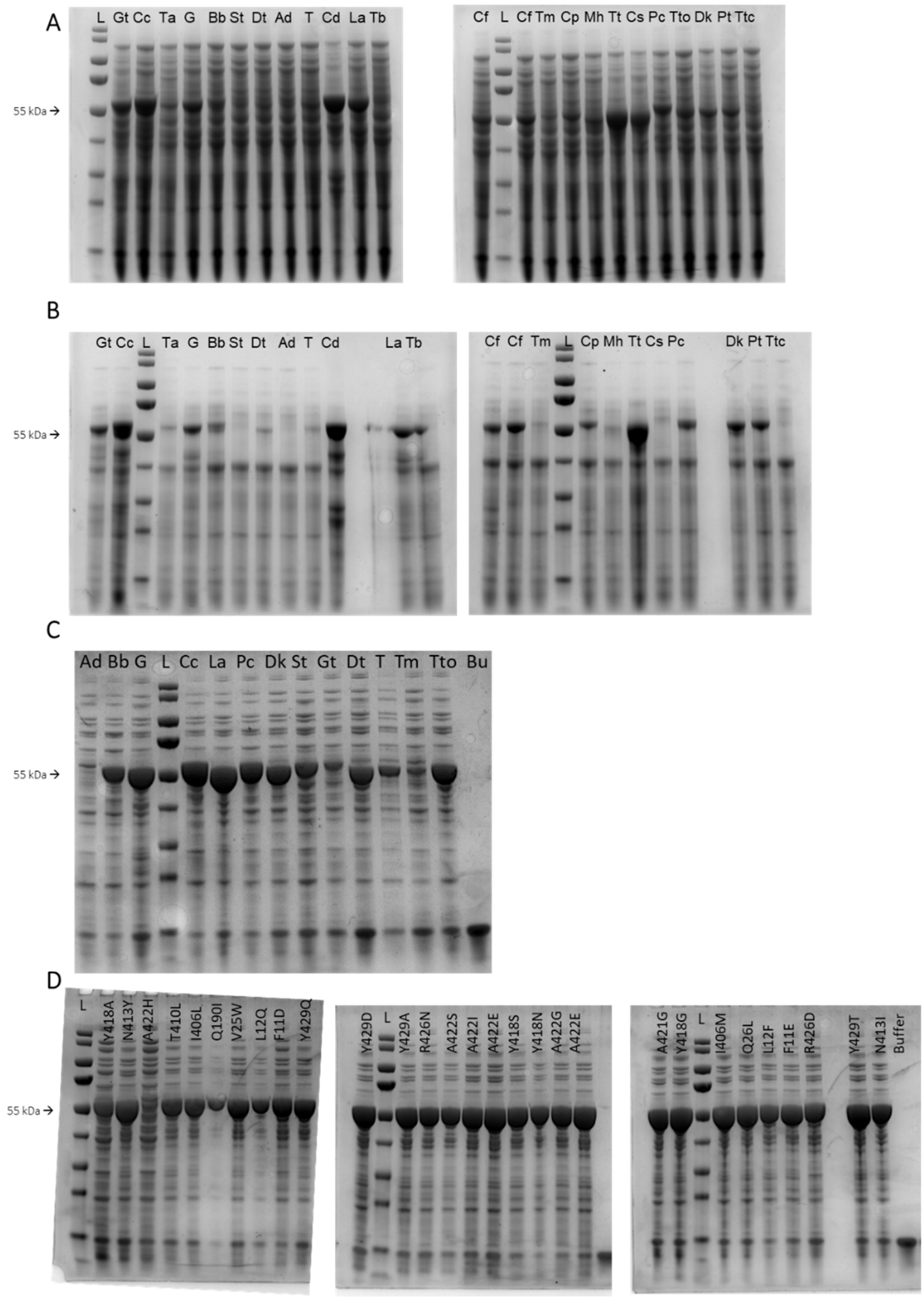

Figure S1. Expression and enzyme production of AspB and homologs. The panels show Coomassie-InstantBlue-stained SDS-PAGE gels of *E. coli* cell-free extracts loaded before or after heat treatment. All variants have a predicted MW of ca. 55 kDa. Labels as in Table S2; L, Marker protein ladder. Panel **A.** Extracts of *E. coli* showing expression of AspB homologs using pBAD, no heat treatment. **B.** Idem, extracts after heating at 60 °C for 30 min. **C.** Idem, best AspB homologs identified by synthesis assays and homologs not obtained with pBAD expressed using the pET vector and *E. coli* BL21(DE3) cells. Bu, buffer with lysozyme. **D**. Heat-treated cell-free extract with AspB-A5 single mutants mutated around the catalytic SS loop.

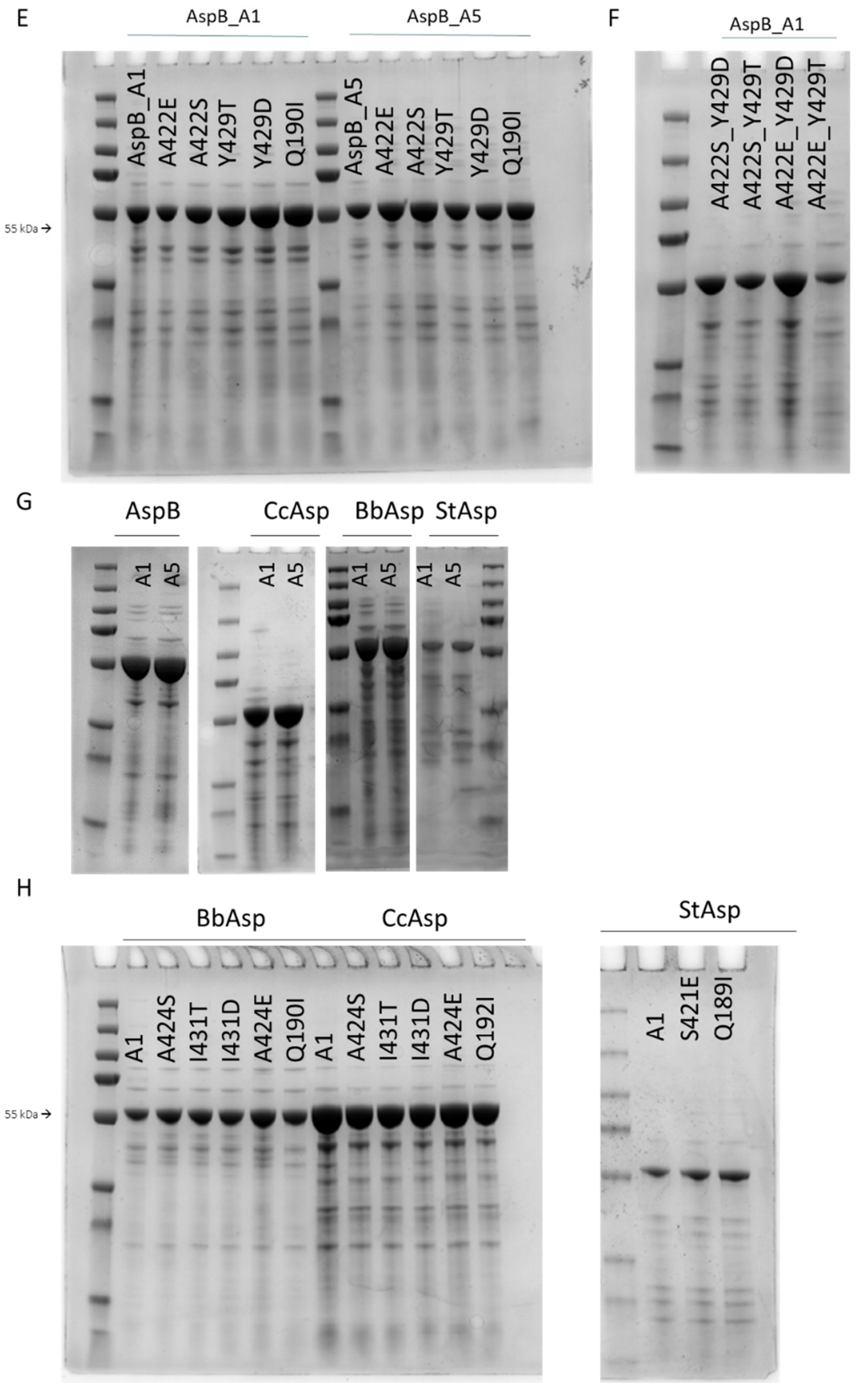

Figure S1-continued. E. Heat-treated extracts of AspB-A1 and AspB-A5 derived variants with mutations in the loop region. **F.** Idem, of variants with loop mutations around the AspB-A1 pocket. **G.** Idem, of AspB homologs carrying A1- and A5-mutations in the substrate binding pockets. **H.** Idem, variants with combined loop mutations and the A1 pocket.

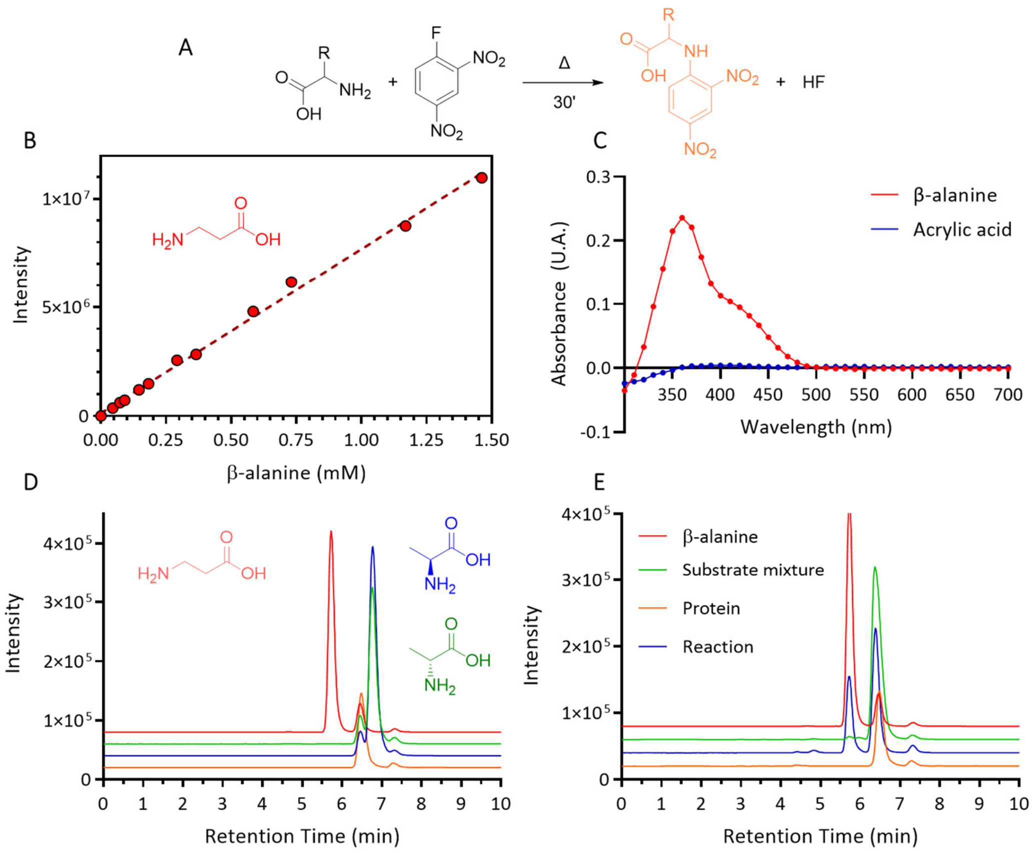

Figure S2. Analysis of β-alanine formation by DNFB derivatization and HPLC. A, Reaction scheme for the derivatization of amino acids with DNFB. **B**, Calibration curve for quantification of DNFB-derivatized β-alanine, detection range 25 µM - 1.3 mM (in assay). **C**, UV-visible spectrum of derivatized β-alanine (red) or acrylic acid (blue). **D**, Chromatograms of standards with DNFB: β-alanine (red), L-α-alanine (blue), D-α-alanine (green) and milliQ water as control (orange). **E**, Chromatographic separation of components in a reaction mixture incubated with AspB as catalyst: β-alanine standard (red), substrate mixture without protein (blue), protein incubated without substrate (orange) and small-scale reaction (green). Retention time β-alanine 5.8 min.

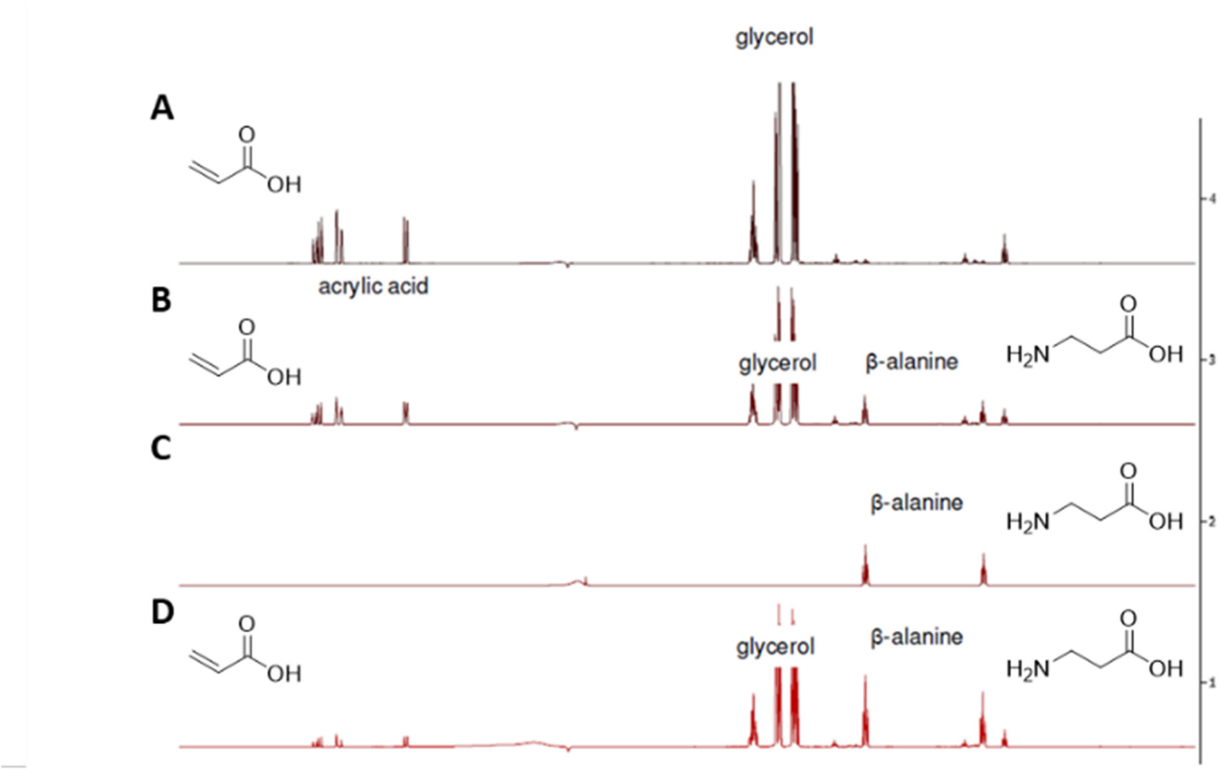

Figure S3. Identification by ^{1}H-NMR of β-alanine formed by AspB-A5 as catalyst. ^{1}H-NMR analysis in $CDCl_3$ at 400 MHz of reaction mixture with: **A**, acrylic acid, **B**, acrylic acid and β-alanine, **C**, standard analysed without glycerol and **D**, reaction performed by AspB-A5 after 24h.

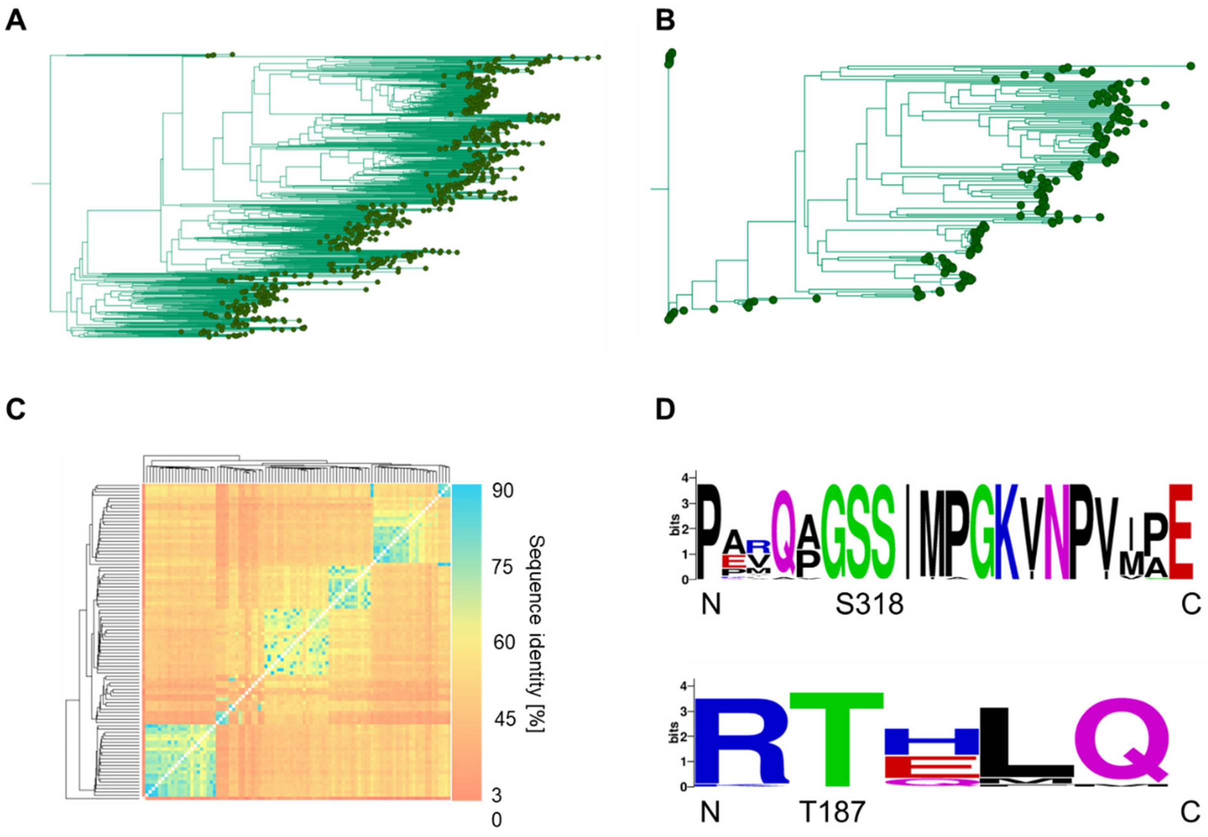

Figure S4. Genome mining approach for finding AspB homologs. The amino acid sequence of AspB was used as a query for the search for homologs in the genomes of thermophilic organisms and metagenomes. **A**, Cladogram of the initial >1450 sequences. **B**, Sequences (446 total) remaining after a sequence identity cut-off of 30 and 90%. **C**, Remaining sequences (97) after removing aberrant and redundant sequences, ordered in an identity matrix aiming to find diverse homologs. **D,** Schematic representation of the conservation of the serine-serine catalytic loop (upper panel), and the conserved threonine from the α-carboxylate binding site (lower panel) together with the surrounding sequence of the selected proteins.

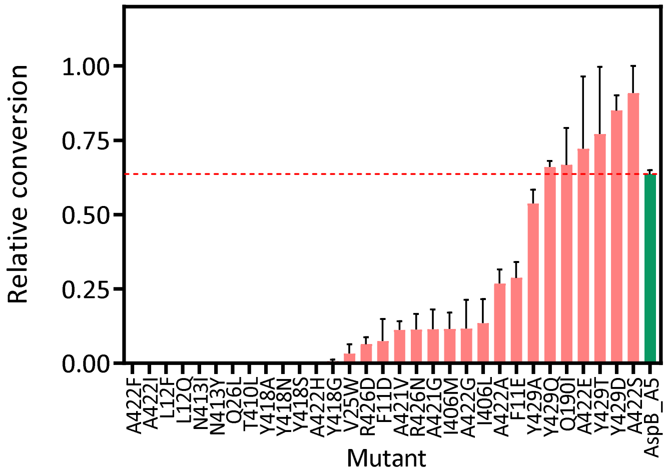

Figure S5. Conversion of acrylic acid to β-alanine by AspB-A5 loop region mutants under standard reaction conditions. Two mutants show significantly better conversion in comparison to AspB-A5.

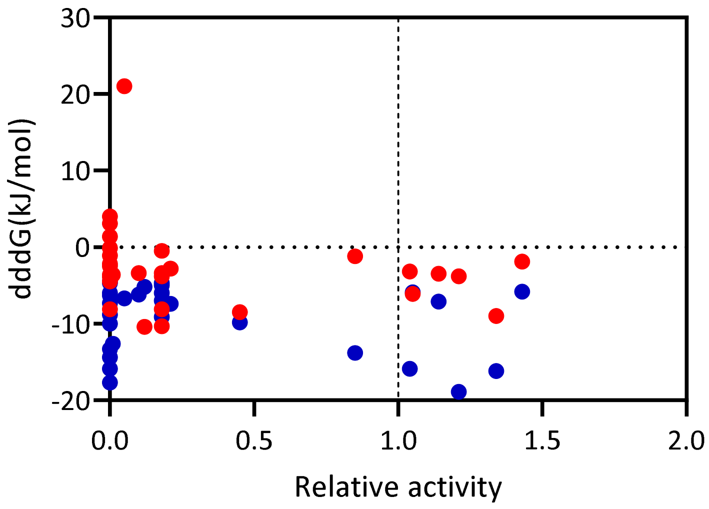

Figure S6. **Comparison of Rosetta and FoldX calculations with experimentally observed effects on activity**. The average of the relative activities of the variants in comparison to AspB-A5 are shown, the activity is compared with FoldX (in red) and Rosetta (in blue) calculations.

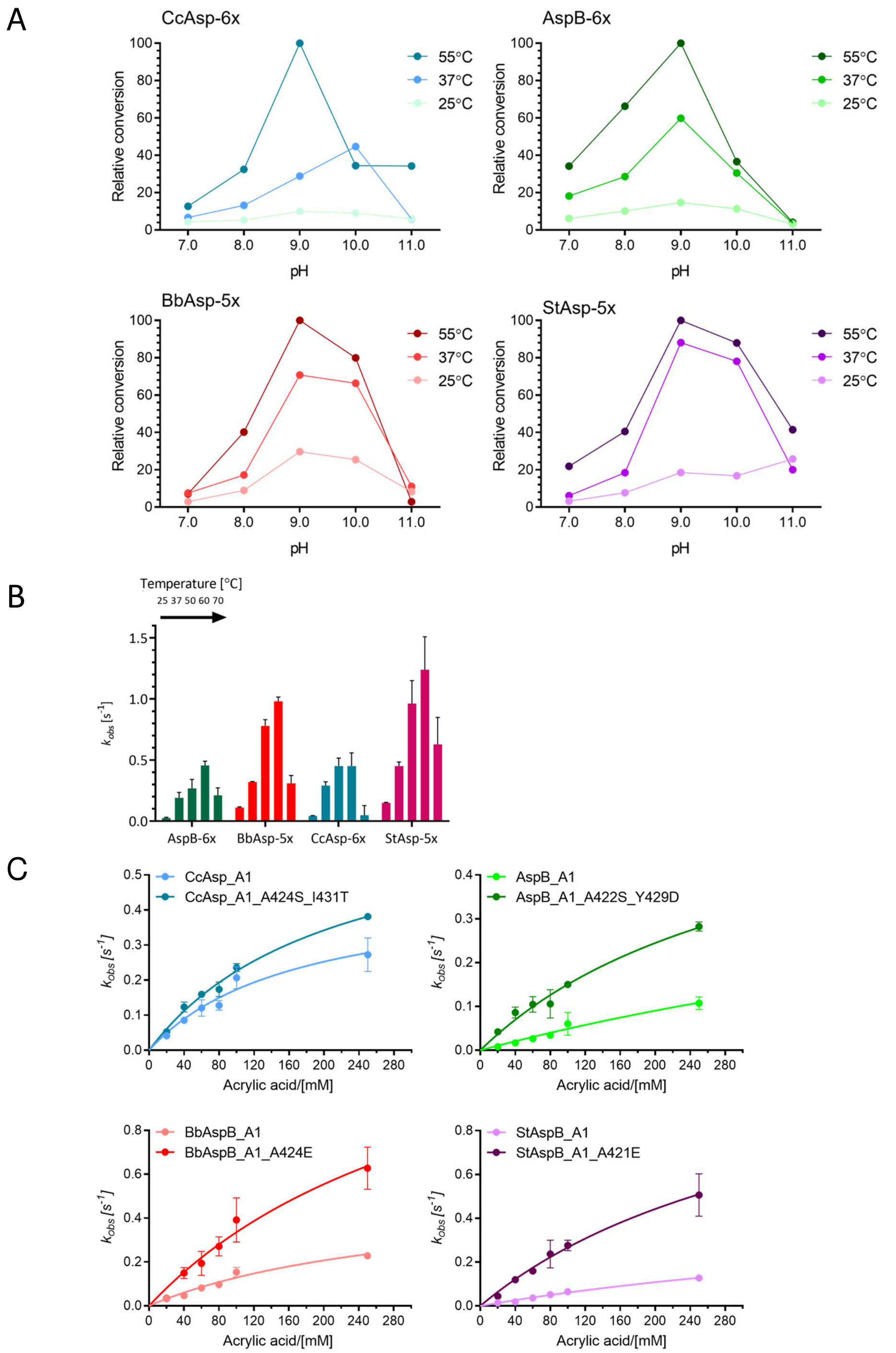

Figure S7. **Activity profiling of engineered aspartase variants**. Activity at varying conditions of four aspartase variants with removed α-carboxylate binding pockets and closed-loop stabilizing mutations. Labelling: CcAsp variants in blue, AspB variants in green, BbAsp variants in red and StAsp variants in purple. Panels: **A**, pH-dependent conversion profiles at three different temperatures. Data are from single measurments. **B**, Temperature-activity profiles. Data are averages of duiplicates. **C**, Initial rates of β-alanine synthesis at 37°C at varying acrylic acid concentrations for the best 4 variants containing loop-stabilizing mutations, in comparison to the respective parents without loop-stabilizing mutations. The affinities for acrylic acid are rather low, but in some cases saturation is approached at 250 mM. Data are averages of duplicates.

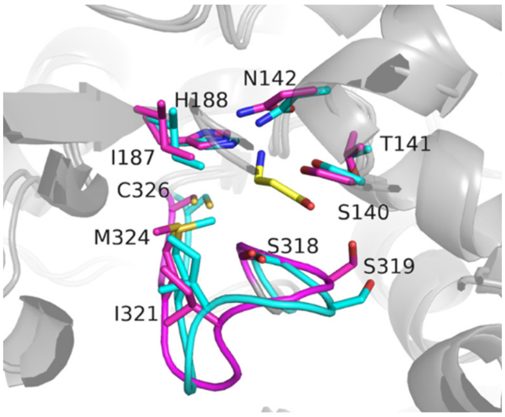

Figure S8. Comparison of a Rosetta-designed AspB variant with the SS-loop in a closed conformation (cyan) and the crystallographic structure of AspB-6x (magenta). Side chains are shown as sticks. The β-alanine used to design the variants is shown as yellow sticks.

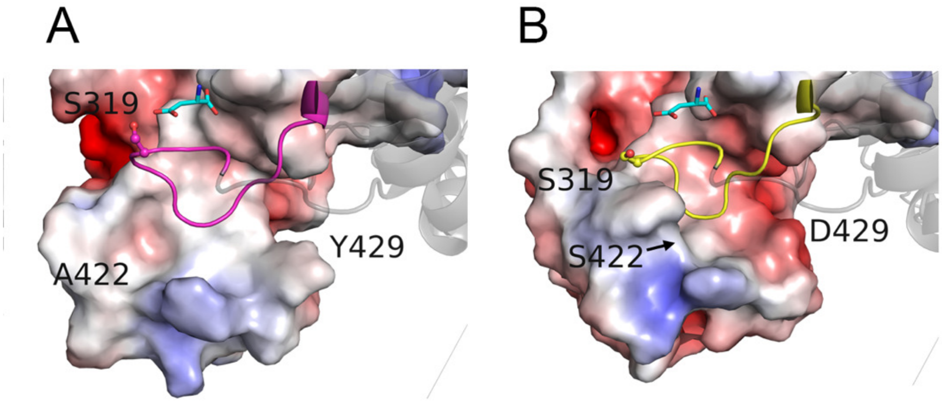

Figure S9. **Effect of the introduced closed-conformation inducing mutations of AspB-6x and wild-type AspB**. Panels: **A**. Electrostatic surface of wild-type AspB in the closed-loop conformation. Residues Tyr429 and Tyr418 (yellow arrow) "release" the loop by rotating away and the C-terminal domain slightly changes its conformation to cover the hydrophobic pa tch, that is still partially exposed. Bound L-Asp represented as sticks (cyan). **B**. Electrostatic surface of AspB-6x variant. Mutations A422S and Y429D redistribute the surface charges and the C-terminal domain adopts a different conformation. L-Asp (cyan) is superimposed for reference. Subunit B is not shown for clarity.
